# Long-term adherence to adapted physical activity in patients with chronic inflammatory arthritis: Insights from a longitudinal observational study

**DOI:** 10.1371/journal.pone.0349043

**Published:** 2026-05-20

**Authors:** Hugo Gondouin, Mathias Poussel, Eliane Albuisson, Margaux Temperelli, Oriane Hily, Anthony Moussu, Isabelle Chary-Valckenaere, Damien Loeuille, Bruno Chenuel, Edem Allado

**Affiliations:** 1 CHRU-Nancy, University Center of Sports Medicine and Adapted Physical Activity, Nancy, France; 2 CHRU-Nancy, Département de Rhumatologie, Université de Lorraine, Nancy, France; 3 DevAH, Université de Lorraine, Nancy, France; 4 CHRU -Nancy, Direction de la Recherche Clinique et de l’Innovation, UMDS, Nancy, France; 5 CNRS, IECL, Université de Lorraine, Nancy, France; 6 Département du Grand Est de Recherche en Soins Primaires: DEGERESP, Université de Lorraine, Nancy, France; 7 IMoPA, Université de Lorraine, Nancy, France; Kyoto University Graduate school, JAPAN

## Abstract

Adapted physical activity is recommended as a non-pharmacological strategy for the management of chronic inflammatory arthritis. However, the long-term adherence of patients to physical activity programs remains a major challenge in clinical practice. The present study aimed to assess adherence to World Health Organization (WHO) physical activity recommendations and to identify factors associated with long-term adherence in patients with chronic inflammatory arthritis (CIA).

This longitudinal observational study included patients with spondyloarthritis, psoriatic arthritis, or rheumatoid arthritis who were referred to a specialized center for adapted physical activity between 2019 and 2021. Baseline demographic, clinical, and treatment-related variables were collected during the initial medical consultation. In August 2023, participants were contacted by telephone to assess their level of physical activity using a standardized questionnaire. Adherence was defined as meeting the WHO recommendations of at least 150 minutes of moderate-intensity physical activity or 75 minutes of vigorous physical activity per week.

Among the 78 patients initially included, 61 responded to the follow-up assessment. Of these, 41 participants (67.2%) met the WHO physical activity recommendations. In univariate analyses, no clinical or treatment-related variables were significantly associated with long-term adherence. Male sex was the only factor significantly associated with adherence, with men showing a higher likelihood of meeting the WHO recommendations after adjustment for underlying pathology (OR 7.85, 95% CI 1.60–38.42).

In this real-world cohort of patients with chronic inflammatory arthritis, approximately two-thirds of respondents maintained a level of physical activity consistent with WHO recommendations more than one year after the initial consultation. Clinical disease characteristics were not associated with adherence, while sex appeared to influence long-term engagement in physical activity. These findings highlight the need for further studies to better understand determinants of long-term adherence to adapted physical activity in this population.

## Introduction

Chronic inflammatory arthritis (CIA), including rheumatoid arthritis (RA), spondyloarthritis (SpA), and psoriatic arthritis (PsA), are chronic systemic diseases primarily affecting the joints and entheses. These conditions represent an important public health concern due to their impact on pain, physical function, and long-term disability. Their estimated prevalence in the general population is approximately 1% for RA, 0.43% for SpA, and between 0.05% and 0.3% for PsA [1–3].

Alongside pharmacological treatments, non-pharmacological interventions are now recognized as an essential component of CIA management. In particular, physical activity is recommended as part of comprehensive care for patients with inflammatory arthritis [4]. Regular physical activity has been associated with numerous health benefits in the general population, including reduced cardiovascular risk, improved functional capacity, and better overall health outcomes [5,6].

In patients with CIA, adapted physical activity (APA) has been shown to improve muscle strength [7–12], functional capacity [13–15], bone mineral density [16], aerobic capacity [17], sleep quality, and fatigue [18–22]. Physical activity may also contribute to reducing disease activity and improving quality of life [9,10,23–28]. For these reasons, international recommendations, including those from the European Alliance of Associations for Rheumatology (EULAR), strongly encourage patients with inflammatory arthritis to engage in regular physical activity adapted to their condition [4].

Despite these well-documented benefits, maintaining regular physical activity over time remains challenging for many patients with chronic diseases. Adherence to long-term therapeutic strategies is frequently suboptimal, with non-adherence rates approaching 50% [29]. Several studies have reported that a large proportion of patients with arthritis do not meet recommended physical activity levels. For example, approximately 60% of patients with arthritis do not follow physical activity recommendations [30], and only about 29% of patients with inflammatory joint diseases meet current guidelines [31]. Similar findings have been reported in specific CIA populations. In axial SpA, adherence to EULAR physical activity recommendations has been estimated at around 46.9% [32–35]. In patients with RA, studies have reported lower physical activity levels compared with healthy individuals, although reported adherence rates vary widely across studies, [36–38].

Long-term adherence to exercise programs therefore represents a major challenge in the management of inflammatory arthritis. Previous research has shown that adherence to home exercise programs tends to decline progressively over time. In patients with ankylosing spondylitis, Hidding et al. reported that adherence to prescribed exercise programs decreased from 86% during the first six weeks to 63% over the following nine months and 51% during the subsequent nine months [39–41].

Although the benefits of physical activity in CIA are well established, fewer studies have explored the determinants of long-term adherence to adapted physical activity in routine clinical practice. Identifying factors associated with sustained engagement in physical activity may help clinicians better support patients and optimize rehabilitation strategies.

Therefore, the aim of this study was to assess adherence to World Health Organization (WHO) physical activity recommendations and to identify factors associated with long-term adherence to adapted physical activity in patients with chronic inflammatory arthritis referred to a specialized clinical center.

## Methods

### Study design

This longitudinal observational study aimed to assess long-term adherence to APA and to identify factors associated with adherence among patients with chronic inflammatory arthritis.

### Setting

The study was conducted at the University Center of Sports Medicine and Adapted Physical Activity at Nancy University Hospital (France), a tertiary referral center providing specialized consultations for patients with chronic diseases requiring evaluation and prescription of adapted physical activity as part of their clinical management.

### Participants

Patients were eligible if they were aged 18 years or older and had a diagnosis of chronic inflammatory arthritis, including spondyloarthritis (SpA), rheumatoid arthritis (RA), or psoriatic arthritis (PsA). Participants were included if they were referred to the specialized APA consultation between February 1, 2019 and December 31, 2021 and received a prescription for adapted physical activity during this consultation. Patients were consecutively included during the study period. No specific APA program was defined according to the type of inflammatory arthritis.

### Data collection and variables

Baseline demographic, clinical, and treatment-related data were extracted from medical records at the time of the initial consultation. Demographic variables included age, sex, and body mass index (BMI). Disease-related variables included the type of inflammatory arthritis and disease duration. Clinical assessments recorded at baseline included the Bath Ankylosing Spondylitis Functional Index (BASFI), the Bath Ankylosing Spondylitis Disease Activity Index (BASDAI), and the Disease Activity Score in 28 joints (DAS28).

Information on treatments was also collected, including biological disease-modifying antirheumatic drugs (bDMARDs), conventional disease-modifying antirheumatic drugs (cDMARDs), nonsteroidal anti-inflammatory drugs (NSAIDs), and analgesics. Socio-professional status was also recorded and categorized into six groups: executives and intermediate professions, farmers and craftsmen, employees or workers, retirees or unemployed individuals, students, and individuals receiving disability benefits.

### Intervention and outcomes

During the initial consultation, patients underwent a medical evaluation including clinical assessment and counseling regarding adapted physical activity. Based on this evaluation, individualized recommendations for physical activity were provided in accordance with the WHO physical activity guidelines.

Patients were advised either to initiate adapted physical activity autonomously or to begin with supervised sessions conducted by adapted physical activity instructors or physiotherapists before transitioning to independent practice.

Follow-up evaluation was conducted in August 2023, at least 20 months after the initial consultation. Participants were contacted by telephone and completed a standardized questionnaire assessing their level of physical activity and disease evolution.

Adherence to adapted physical activity was defined according to WHO recommendations as performing at least 150 minutes of moderate-intensity physical activity per week or at least 75 minutes of vigorous-intensity physical activity per week [42]. Participants meeting these criteria were classified as APA adherent, whereas those not meeting these recommendations were classified as APA non-adherent.

### Statistical analysis

Descriptive and comparative analyses were performed according to the type and distribution of the variables. Qualitative variables were expressed as frequencies and percentages, while quantitative variables were presented as mean ± standard deviation (SD) or median and interquartile range (IQR) when appropriate.

Comparisons between APA-adherent and APA-non-adherent participants were performed using Student’s t-test or the Mann–Whitney U test depending on the distribution of the variables. Categorical variables were compared using the Chi-square test or Fisher’s exact test when appropriate.

To identify factors associated with adherence to adapted physical activity, a multivariable logistic regression analysis was conducted. Variables identified in univariate analyses with a significance level of p ≤ 0.10, as well as clinically relevant variables, were considered for inclusion in the model. The final model was adjusted for the underlying pathology.

Given the exploratory nature of the analyses, no correction for multiple comparisons was applied and p-values should therefore be interpreted descriptively. All statistical analyses were performed using IBM SPSS Statistics version 23, with statistical significance defined as p < 0.05.

### Ethics and dissemination

The study was registered with the Information Technology and Freedoms Commission of Nancy University Hospital (IRB number: 2021PI191) and on ClinicalTrials.gov (NCT05146544). The protocol was approved by the Ethics Committee of Nancy University Hospital (reference number 336, chaired by Professor Martinet) and conducted in accordance with the principles of the Declaration of Helsinki. As this was an observational study of routine clinical practice and no physically or psychologically invasive intervention was performed, verbal informed consent was obtained from all participants for the use of their medical data for research purposes. The information provided to each patient and the verbal consent obtained were recorded in the medical record at that time.

### Patient and public involvement

Patients and members of the public were not involved in the design, conduct, reporting, or dissemination of this research.

## Results

### Participant characteristics

**Fig 1:**
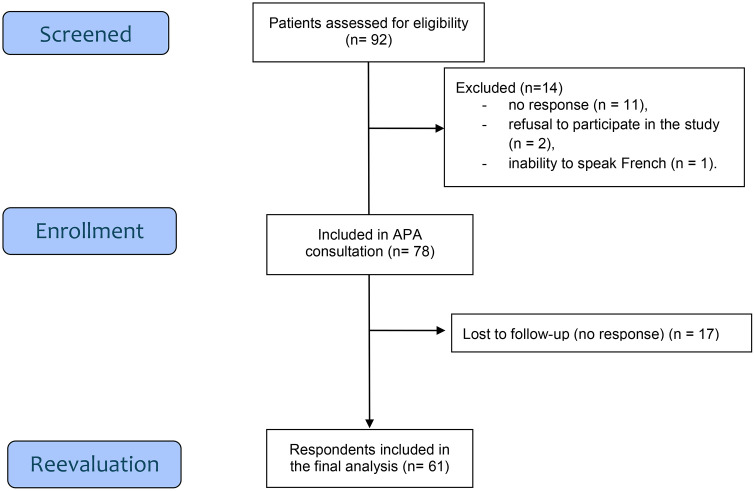
Flow chart of patient inclusion and follow-up.

A total of 92 patients were initially eligible for the study, of whom 78 were included following referral to the specialized consultation for adapted physical activity (Fig 1). Among these participants, 61 responded to the follow-up assessment conducted in August 2023 and were included in the final analysis, corresponding to a response rate of 78.2%.

Baseline demographic and clinical characteristics of the study population are presented in Table 1. The majority of participants were women (69.2%), with a mean age of 44.4 ± 12.0 years and a mean body mass index of 29.9 ± 8.0 kg/m². Most patients were diagnosed with SpA (65.4%), followed by PsAs (23.1%) and RA (11.5%).

**Table 1:** Baseline demographic and clinical characteristics (n = 78).

	Total (n = 78)
Women	54 (69.2)
Age (years)	44.4 (*±*12.0)
Body mass index, kg/m² • Height (cm) • Weight (kg)	29.9 (*±*8.0) 167.7 (*±*8.4) 83.8 (*±*22.1)
Rheumatologic disease • Spondyloarthritis (SpA) • Psoriasic arthritis (PsA) • Rheumatoid arthritis (RA)	51 (65.4) 18 (23.1) 9 (11.5)
Rheumatologic disease duration (years) • Spondyloarthritis (SpA) • Psoriasic arthritis (PsA) • Rheumatoid arthritis (RA)	9.2 (7.4) 8.8 (7.1) 7.7 (5.3) 14.1 (11.1)
Questionnaires baseline • BASFI (n = 58) • BASDAI (n = 49) • DAS28 (n = 10)	48.3 (*±*27.4) 5.6 (*±*2.2) 2 (*±*1.6)
Baseline biotherapy treatment (n = 75) • No treatment (No bDMARDs) • Treatment (bDMARDs, current) • Treatment history (bDMARDs, previous)	18 (24.0) 40 (53.3) 17 (22.7)
Socio-professional work (n = 71) • Executives / intermediate professions • Farmers / craftsmen, • Employees / workers • Retirees / unemployed, others • Students • Invalidity	12 (16.9) 3 (4.2) 26 (36.6) 16 (22.5) 2 (2.8) 12 (16.9)

Data are presented as n (%) for categorical variables, mean ± SD for normally distributed continuous variables, and median [interquartile range] for non-normally distributed variables.

The mean disease duration was 9.2 ± 7.4 years. Patients with RA had a longer mean disease duration (14.1 ± 11.1 years) compared with those with SpA (8.8 ± 7.1 years) and PsA (7.7 ± 5.3 years).

Baseline clinical assessments included BASFI (mean 48.3 ± 27.4), BASDAI (mean 5.6 ± 2.2), and DAS28 (mean 2.0 ± 1.6). Regarding treatments, 53.3% of participants were receiving bDMARDs at baseline, whereas 24.0% had no biological treatment and 22.7% had previously received bDMARDs that were no longer administered at the time of the consultation.

Socio-professional categories were mainly represented by employees or workers (36.6%), followed by retirees or unemployed individuals (22.5%) and executives or intermediate professions (16.9%).

### Adherence to adapted physical activity

Among the 61 participants who completed the follow-up assessment, 41 (67.2%) met WHO physical activity recommendations and were classified as APA adherent, whereas 20 (32.8%) did not meet the recommended level of physical activity and were classified as APA non-adherent.

Walking was the most frequently reported physical activity (77.0%), followed by cycling (13.1%). The mean daily sedentary time reported by participants was 4.6 ± 2.4 hours, with a maximum reported value of 13 hours.

### Factors associated with adherence to adapted physical activity

Comparisons between APA-adherent and APA-non-adherent participants are presented in Table 2. No statistically significant differences were observed between the two groups regarding age (42.5 ± 9.9 vs. 46.2 ± 9.7 years; p = 0.172), BMI (31.8 ± 11.8 vs. 29.8 ± 6.3 kg/m²; p = 0.534), disease duration, baseline clinical scores (BASFI, BASDAI, DAS28), sedentary behavior, or treatments.

**Table 2:** Demographic and clinical characteristics related to adherence to adapted physical activity.

	APA no adherent (APA -) (n = 20)	APA adherent (APA +) (n = 41)	p-value
Women	18 (90.0)	22 (53.7)	**0.005**
Age, years	42.5 (9.9)	46.2 (9.7)	0.172
Body mass index, kg/m^2^	31.8 (11.8)	29.8 (6.3)	0.534
Rheumatologic disease duration, years • Spondyloarthritis (SpA) • Psoriasic arthritis (PsA) • Rheumatoid arthritis (RA)	6.6 (4.9) 6.7 (3.1) 6.3 (3.1)	9.9 (8.4) 8.6 (6.8) 25.3 (9.8)	0.151 0.530 0.068
Questionnaires baseline • BASFI • BASDAI • DAS28	47.5 (28.8) 5.7 (1.8) 3.1 (1.6)	51.7 (26.4) 5.4 (2.2) 2.0 (1.8)	0.669 0.771 0.384
Sedentary behaviour, hours	4.8 (2.5)	4.6 (2.4)	0.727
Autonomous APA practice (baseline)	4 (20.0)	8 (19.5)	0.608
Baseline biotherapy treatment (n = 60) • No treatment (No bDMARDs) • Treatment (bDMARDs, current) • Treatment history (bDMARDs, previous)	6 (30.0) 10 (50.0) 4 (20.0)	9 (22.5) 23 (57.1) 8 (20.0)	0.804
Baseline biotherapy treatment (n = 60) • No treatment (No bDMARDs and bDMARDs, previous) • Treatment (bDMARDs current)	10 (50.0) 10 (50.0)	17 (42.5) 23 (57.1)	0.582
Daily pain (Visual analogic scale (VAS)), mm	54.3 (23.1)	56.3 (18.8)	0.807
Joint pain since the beginning of the APA • Reduction • Stable • Increase	5 (25.0) 7 (35.0) 8 (40.0)	13 (31.7) 17 (41.5) 11 (26.8)	0.578
Analgesic • Reduction • Stable • Increase	3 (15.0) 13 (65.0) 4 (20.0)	4 (9.8) 18 (43.9) 19 (46.3)	0.137
Socio-professional work • Executives / intermediate professions • Farmers / craftsmen, • Employees / workers • Retirees / unemployed, others • Students • Invalidity	2 (11.8) 2 (11.8) 5 (29.4) 3 (17.6) 1 (5.9) 4 (23.5)	9 (22.5) 1 (2.5) 16 (40.0) 8 (20.0) 0 (0.0) 6 (15.0)	0.316

Legend: Data are presented as n (%) for categorical variables, mean ± SD for normally distributed continuous variables, and median [interquartile range] for non-normally distributed variables. Group comparisons between APA-adherent and APA non-adherent participants were performed using Student’s t-test or the Mann–Whitney U test for continuous variables, and the Chi-square test or Fisher’s exact test for categorical variables.

Sex was the only factor significantly associated with APA adherence. Women represented 90.0% of the APA non-adherent group compared with 53.7% of the APA adherent group (p = 0.005). In univariate analysis, being male was associated with a higher likelihood of meeting WHO physical activity recommendations (OR 7.78, 95% CI [1.59–37.91]). This association remained significant after adjustment for the underlying pathology in the multivariable logistic regression analysis (OR 7.85, 95% CI [1.60–38.42]).

No other demographic, clinical, or treatment-related variables were significantly associated with APA adherence.

## Discussion

This longitudinal observational study assessed long-term adherence to APA in patients with CIA referred to a specialized consultation. Overall, approximately two-thirds of respondents met WHO physical activity recommendations more than one year after the initial consultation. Among the variables explored, male sex was the only factor significantly associated with APA adherence, whereas no significant associations were observed with demographic, clinical, or treatment-related variables.

Regular physical activity is widely recognized as a cornerstone of non-pharmacological management in inflammatory arthritis. Exercise interventions have consistently been shown to improve muscle strength, physical function, aerobic capacity, and quality of life in patients with CIA without worsening disease activity [7–17]. In addition, physical activity has been associated with improvements in fatigue, sleep quality, and psychological well-being in this population [18–28]. Based on this evidence, international recommendations, including those from EULAR, encourage patients with inflammatory arthritis to engage in regular physical activity adapted to their functional capacity and disease status [4].

Despite these well-established benefits, adherence to physical activity recommendations remains suboptimal in patients with arthritis. Previous studies have reported that a large proportion of patients with inflammatory joint diseases do not meet recommended levels of physical activity. For example, approximately 60% of patients with arthritis do not follow physical activity recommendations [30], and only around 29% of patients with inflammatory joint diseases achieve recommended activity levels [31]. Similarly, studies conducted in axial SpA populations have reported adherence rates to physical activity recommendations of approximately 46.9% [32–35]. In RA populations, physical activity levels are also frequently lower than those observed in healthy individuals [36–38].

In this context, the adherence rate observed in the present study appears relatively encouraging, with approximately two-thirds of respondents reporting levels of physical activity consistent with WHO recommendations. Although comparisons between studies should be interpreted cautiously due to differences in populations and measurement methods, this finding suggests that specialized consultations dedicated to APA may contribute to promoting sustained engagement in physical activity among patients with CIA.

Nevertheless, maintaining long-term engagement in physical activity remains challenging. Previous studies have shown that adherence to exercise programs tends to decline progressively over time. In patients with ankylosing spondylitis, Hidding et al. demonstrated that adherence to prescribed exercise programs decreased from 86% during the first six weeks to 63% over the following nine months and 51% during the subsequent nine months [39–41]. These findings highlight the difficulty of sustaining behavioral changes related to physical activity and underline the importance of identifying factors influencing long-term adherence.

In the present study, male sex was the only factor significantly associated with APA adherence. Men were more likely than women to meet WHO physical activity recommendations at follow-up. Similar sex differences in physical activity participation have been described in the general population. However, this result should be interpreted cautiously. The wide confidence interval associated with the odds ratio suggests substantial uncertainty around the estimate, likely reflecting the relatively small sample size and the imbalance in sex distribution within the cohort. Larger studies are therefore required to determine whether sex represents an independent determinant of long-term adherence to APA in patients with CIA. These findings may also suggest that tailored strategies to support physical activity engagement among women could be beneficial.

No significant associations were observed between APA adherence and disease-related variables such as disease duration, disease activity scores, or ongoing treatments. These findings suggest that clinical disease characteristics alone may not fully explain patients’ engagement in physical activity. However, the absence of statistically significant associations should not be interpreted as evidence of the absence of an effect. Given the relatively limited sample size, the study may have been underpowered to detect small-to-moderate associations between clinical factors and APA adherence.

Several limitations should be considered when interpreting the results of this study. First, the relatively small sample size may limit statistical power and contribute to the wide confidence intervals observed in the regression analysis. Second, 17 participants did not respond to the follow-up assessment, which may introduce potential attrition bias. As adherence was evaluated only among respondents, the reported adherence rate should therefore be interpreted with caution. Third, the analyses presented in Table 2 involved multiple exploratory comparisons without correction for multiple testing, and the resulting p-values should therefore be interpreted descriptively. Finally, the assessment of physical activity relied on self-reported data collected through a telephone questionnaire, which may be subject to recall bias and social desirability bias.

Despite these limitations, this study also has several strengths. It reflects real-world clinical practice in a specialized consultation dedicated to APA and includes patients with different forms of inflammatory arthritis. In addition, the relatively long follow-up period allowed the evaluation of long-term adherence to physical activity recommendations beyond the initial consultation.

Overall, the results of this study suggest that a substantial proportion of patients with CIA may maintain regular physical activity following an APA consultation. However, identifying determinants of long-term adherence remains challenging. Future studies including larger populations and prospective designs are needed to better understand the factors influencing sustained engagement in physical activity and to develop targeted strategies aimed at improving long-term adherence to APA in patients with inflammatory arthritis.
